# Transcranial random noise stimulation-induced plasticity is NMDA-receptor independent but sodium-channel blocker and benzodiazepines sensitive

**DOI:** 10.3389/fnins.2015.00125

**Published:** 2015-04-10

**Authors:** Leila Chaieb, Andrea Antal, Walter Paulus

**Affiliations:** ^1^Department of Clinical Neurophysiology, Georg-August UniversityGöttingen, Germany; ^2^Department of Epileptology, University of BonnBonn, Germany

**Keywords:** transcranial random noise stimulation (tRNS), transcranial magnetic stimulation (TMS), transcranial direct current stimulation (tDCS), lorazepam (LOR: GABA_*A*_ receptor agonist), ropinirole (ROP: D_2_/D_3_ receptor agonist), carbamazepine (CBZ: sodium channel blocker), dextromethorphan (DMO: NMDA receptor antagonist), D-cycloserine (D-CYC: partial NMDA receptor agonist)

## Abstract

**Background:** Application of transcranial random noise stimulation (tRNS) between 0.1 and 640 Hz of the primary motor cortex (M1) for 10 min induces a persistent excitability increase lasting for at least 60 min. However, the mechanism of tRNS-induced cortical excitability alterations is not yet fully understood.

**Objective:** The main aim of this study was to get first efficacy data with regard to the possible neuronal effect of tRNS.

**Methods:** Single-pulse transcranial magnetic stimulation (TMS) was used to measure levels of cortical excitability before and after combined application of tRNS at an intensity of 1 mA for 10 min stimulation duration and a pharmacological agent (or sham) on eight healthy male participants.

**Results:** The sodium channel blocker carbamazepine showed a tendency toward inhibiting MEPs 5–60 min poststimulation. The GABA_*A*_ agonist lorazepam suppressed tRNS-induced cortical excitability increases at 0–20 and 60 min time points. The partial NMDA receptor agonist D-cycloserine, the NMDA receptor antagonist dextromethorphan and the D_2_/D_3_ receptor agonist ropinirole had no significant effects on the excitability increases seen with tRNS.

**Conclusions:** In contrast to transcranial direct current stimulation (tDCS), aftereffects of tRNS are seem to be not NMDA receptor dependent and can be suppressed by benzodiazepines suggesting that tDCS and tRNS depend upon different mechanisms.

## Introduction

Experience dependent neuronal activity changes lead to a strengthening or weakening of synaptic connections, summarized as neuroplasticity (Bliss and Lomo, 1973; Cooke and Bliss, 2006). Transcranial stimulation by either magnetic (transcranial magnetic stimulation: TMS) or electrical methods (transcranial electrical stimulation: tES) allow for the induction of similar neuroplastic alterations. In human subjects the motor cortex (M1) is a preferred model for studying these effects since the muscle twitch elicited by suprathreshold TMS (motor-evoked-potential: MEP) allows for an easy readout of plasticity alterations by using the MEP amplitude as a biomarker (for a review see: Ziemann et al., 2008).

Transcranial direct current stimulation (tDCS) modulates cortical excitability in a polarity, stimulation intensity and duration dependent way (Nitsche and Paulus, 2000; Batsikadze et al., 2013; Monte-Silva et al., 2013); generally, it was observed anodal stimulation increasing and cathodal stimulation decreasing levels of motor cortical excitability (Nitsche and Paulus, 2000, 2001). This dependency can be strongly modulated by co-application of neuroactive drugs (Liebetanz et al., 2002; Nitsche et al., 2003a; Abbruzzese et al., 2010), e.g., as seen with the dose-dependent reversal of tDCS aftereffects by dopamine (Kuo et al., 2008; Nitsche et al., 2009; Monte-Silva et al., 2010). Transcranial random noise stimulation (tRNS) is a new tES method, which was also shown to induce an increase in sustained levels of cortical excitability in the M1 (Terney et al., 2008; Moliadze et al., 2010, 2012; Chaieb et al., 2011). Stimulation with 1 mA tRNS leads to a persistent elevation of single-pulse TMS elicited MEPs lasting up to 1 h post-stimulation after 10 min tRNS duration. Lower intensities at around 0.4 mA tRNS lead to inhibitory aftereffects comparable to what has been observed with cathodal stimulation (Moliadze et al., 2012).

With regard to the possible underlying mechanisms of these neuroplastic effects it was shown that the electric field generated by tDCS is able to increase or reduce the membrane potential of neurons in a linear way, even in cell types with a spherical dendritic arborization pattern (Radman et al., 2009). With invasive electrodes it has been shown that cellular targets closest to the anode hyperpolarise, while those elements closest to the cathode simultaneously depolarise (Chan et al., 1988). However, the cellular targets of transcranially applied electrical currents include morphologically and functionally distinct networks of interneurons and pyramidal cell neurons (Radman et al., 2009). Animal studies and *in vitro* slice preparation studies have demonstrated that neurons with a non-symmetric dendritic morphology are more likely to be activated by the presence of an applied electric field and that they are more susceptible to the polarity of electric fields (Hern et al., 1962; Chan et al., 1988; Radman et al., 2009). Pharmacological intervention in combination with tDCS has revealed its NMDA receptor dependency (Liebetanz et al., 2002) and further demonstrated the possibility of selectively prolonging anodal aftereffects by applying the indirect NMDA receptor agonist d-cycloserine, as well as amphetamine, and modulating cathodal aftereffects by low dose pergolide, a dopamine receptor agonist (Nitsche et al., 2004a,b, 2006; Monte-Silva et al., 2009). Both carbamazepine, a voltage-gated sodium channel blocker and flunarizine, a calcium channel antagonist, abolished the short-duration aftereffects induced by anodal tDCS, but not by cathodal tDCS (Liebetanz et al., 2002; Nitsche et al., 2003b). This suggests that the mechanisms underlying tDCS are ion-channel dependent, selectively affecting neurons and generating inhibitory and excitatory modulations in cortical excitability, producing, respectively long-term depression (LTD) and long-term potentiation (LTP)-like effects. Here, we have implemented this combined approach for tRNS in order to gain a better understanding of its underlying mechanisms. This experiment was planned as a pilot study providing first efficacy data. We administered five pharmacological agents in order to characterize essential receptors and ion channels possibly involved in the generation of tRNS aftereffects: lorazepam (LOR: GABA_*A*_ receptor agonist), ropinirol (ROP: dopamine receptor 2/3 agonist), carbamazepine (CBZ: a sodium channel blocker), dextromethorphan (DMO: NMDA receptor antagonist) and D-cycloserine (D-CYC: partial NMDA receptor agonist).

## Methods

### Subjects

Eight healthy male subjects (mean age 30.1 ± 5.2 years) participated in the study. In order to estimate the numbers of subjects needed, power analysis were done based on the results of previously published data (Liebetanz et al., 2002; Huang et al., 2007). In order to detect the difference in the mean MEP size between PLC + tES and a given drug condition + tES with 95% confidence and 80% power, min. Seven subjects should be included. All participants were informed as to all aspects of the experiments and gave written consent. None of the participants suffered from any neurological or psychological disorders, nor had any metal implants or implanted devices, took any relevant medication regularly or prior to their participation. Seven of the subjects were right-handed according to the Edinburgh handedness inventory (Oldfield, 1971). All aspects of the protocol conformed to the Declaration of Helsinki and were approved by the Ethics Committee of the Medical Faculty of the University of Göttingen.

### Transcranial random noise stimulation

tRNS was delivered by a battery-driven electrical stimulator (Version DC-Stimulator-Plus, NeuroConn GmbH, Ilmenau, Germany) through conductive-rubber electrodes, placed in two saline-soaked sponges. In the stimulation mode “noise” a random level of current is generated for every sample (sampling rate 1280 samples per second). The random frequencies are normally distributed; the probability density function follows a bell-shaped curve. In the frequency spectrum all coefficients have a similar size (“white noise”). The noise signal contains all frequencies up to half of the sampling rate, i.e., a maximum of 640 Hz (Terney et al., 2008). Due to the statistical characteristics the signal has no DC offset, provided that the offset is set to zero.

The stimulation electrode was placed over the left M1, which was determined prior to stimulation by single-pulse TMS (see below). The return electrode was placed over the contralateral orbit. The size of the stimulation electrode was 4 × 4 cm and the return electrode was 6 × 14 cm. The electrodes were fixated to the head using elastic bands. tRNS was applied for 10 min with a current strength of 1 mA.

### Measuring corticospinal excitability

To detect current-driven changes of excitability, motor evoked potentials (MEPs) of the right abductor digiti minimi muscle (ADM) were recorded following stimulation of its motor-cortical representation field by single-pulse TMS. MEPs were induced using a Magstim 200 magnetic stimulator (Magstim Company, Whiteland, Wales, UK) with a figure-of-eight standard double magnetic coil (diameter of one winding, 70 mm; peak magnetic field, 2.2 T; average inductance, 16.35 μH). Surface electromyogram (EMG) was recorded from the right ADM through a pair of Ag-AgCl surface electrodes in a belly-tendon montage. Raw signals were amplified, band-pass filtered (2 Hz–3 kHz; sampling rate, 5 kHz), digitized with a micro 1401 AD converter (Cambridge Electronic Design, Cambridge, UK) controlled by Signal Software (Cambridge Electronic Design, version 2.13), and stored on a personal computer for offline analysis. Complete relaxation was controlled through visual feedback of EMG activity and whenever it was necessary, the subject was instructed to relax. The coil was held tangentially to the skull, with the handle pointing backwards and laterally at 45° from the midline, resulting in a posterior-anterior direction of current flow in the brain. This orientation of the induced electrical field is thought to be optimal for stimulating the pyramidal tract neurons in the wall of the M1; the representation of the new motor cortex according to Rathelot and Strick, from layers 6 through to layer 1 (Rathelot and Strick, 2009). The optimum position (hot-spot) was defined as the site where TMS resulted consistently in the largest MEP in the resting muscle. The site and the coil position were marked with a skin marker on the scalp to ensure that the coil was held in the correct location throughout the experiment.

### Pharmacological interventions

Two hours prior to the beginning of the experimental session, and two and half hours prior to the administration of tRNS, subjects were given either: 100 mg D-cycloserine (D-CYC), 2 mg ropinirol (ROP), 1 mg lorazepam (LOR), 75 mg dextromethorphan (DMO), 300 mg carbamazepine (CBZ) or an equivalent placebo agent (PLC). These types of drugs and their dosages were previously tested in different tDCS studies (Liebetanz et al., 2002; Nitsche et al., 2003b) and were found to have a maximum blood peak level around 2–3 h. All pharmacological agents were orally administered. To avoid cumulative drug effects, all experimental sessions were separated by a 2 week interval. Both the subjects and the experimenter were blinded to the respective pharmacological condition.

### Experimental procedure

The subjects received the combination of tRNS and a given drug in a randomized order. The randomization was done by the coordinating investigator, who had no contact to the subjects. They were seated in a comfortable reclining chair with a mounted headrest throughout the experiments. All experimental sessions were conducted by the same investigator. Resting motor threshold (RMT), active motor threshold (AMT), the intensity to evoke MEP of ~ 1 mV peak-to-peak amplitude and a baseline of TMS-evoked MEPs (40 stimuli) were recorded at 0.25 Hz prior to stimulation. Stimulus intensities (in percentage of maximal stimulator output) of TMS were determined at the beginning of each experiment. RMT was defined as the minimal output of the stimulator that induced a reliable MEP (~50 μV in amplitude) in at least three of six consecutive trials when the ADM muscle was completely relaxed. AMT was defined as the lowest stimulus intensity at which three of six consecutive stimuli elicited reliable MEP (~200 μV in amplitude) in the tonically contracting ADM muscle of participants (contracting at half-maximal contraction of the ADM) (Rothwell et al., 1999). Immediately following stimulation, 40 single test-pulse MEPs were recorded at 0.25 Hz, i.e., approximately 0, 5, 10 min post tRNS and then every 10 min up to 60 min; then at 90, 120, 240 min intervals and finally 24 h post tRNS.

### Calculations and statistics

Any MEPs with EMG artifacts were rejected. MEP amplitude (peak-to-peak) was automatically calculated using the NuCursor programme (IoN, UCL, London, UK) and the mean value (from minimum 35 MEPs) was determined for each timepoint.

The main aim of this study was to get first efficacy data with regard to the possible neuronal effect of tRNS. Therefore, Two-Way repeated measures ANOVAs (DRUG (CBZ, LOR, DMO, D-CYC, ROP) + tRNS vs. tRNS + PLC) × TIME (baseline, 0, 5, 10, 20, 30, 40, 50, 60, 90, 120, min and 24 h post-stimulation) were used to compare a given different drug condition to the tRNS + PLC condition; DRUG and TIME serving as independent variables, while MEP amplitude was the dependent variable. Effects were considered significant if *p* ≤ 0.05. In the case of a significant main effect of DRUG and interaction of TIME and DRUG, a Fischer *post-hoc* test was performed. Student's *t*-test was used to compare baseline raw MEP amplitudes before stimulation with those afterwards within the placebo condition. All data are given as means ± SEM.

## Results

None of the participants reported any side effects of the tRNS and/or medication. Among the baseline MEP values there were no significant differences (*ps* > 0.8).

### Placebo drug condition

As in previous experiments (Terney et al., 2008; Chaieb et al., 2009; Moliadze et al., 2012) tRNS caused an elevation in MEP sizes for 90 min following 10 min of stimulation (*df* = 7, *t* = 2.9–4.65, *p* < 0.05), with a tendency at 0 min (*p* = 0.07) and 30–40 min (*p* = 0.11 and 0.09) that might be due to the lower number of subjects measured in this study.

### Drug interactions

Results of repeated measures ANOVAs conducted for main effects and interactions with a given DRUG and DRUG × TIME indicate that ROP, DMO, and D-CYC (Figures 2, 3, 5) had no significant effect on TMS elicited MEP amplitudes, when compared to the tRNS + PLC condition (Table 1), although DMO showed a light tendency to inhibit MEPs compared to PLC post-stimulation.

**Table 1:** **Results of the statistical analyses (repeated measures of ANOVA) of each pharmacological condition on the effect of average MEP size after stimulation of the M1 with tRNS compared with tRNS + PLC**.

	**DRUG (1,14)**		**TIME (10,14)**		**DRUG × TIME (10,14)**	
	***F***	***p***	***F***	***p***	***F***	***p***
CBZ	4.98	0.007*	2.09	0.03*	1.56	0.11
LOR	3.3	0.09	1.67	0.08	1.85	0.05*
DMO	0.91	0.36	2.88	0.002*	0.47	0.9
D-CYC	2.1	0.17	2.22	0.06	0.87	0.56
ROP	0.7	0.41	3.00	0.001*	0.77	0.66

*The table reports the F and p-values for conditions DRUG and TIME and for the interaction between DRUG × TIME. Asterisks-bold italic values indicate significant results*.

**Figure 1:**
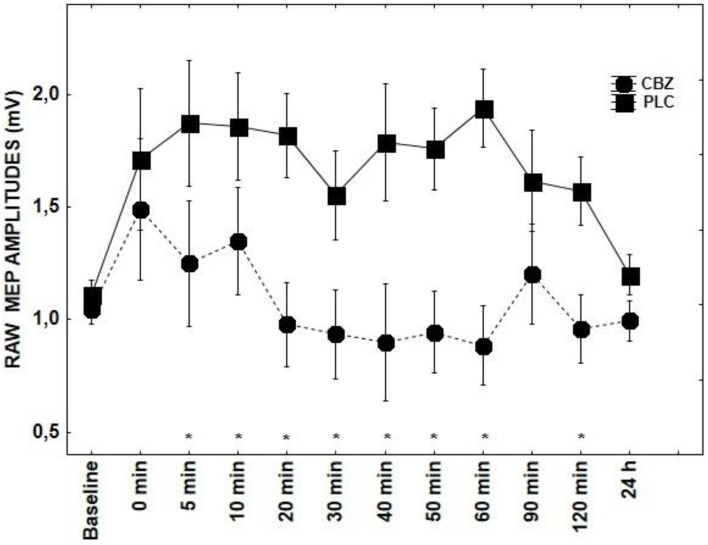
**Shows the effect of CBZ on tRNS-induced aftereffects as measured by averaging TMS-evoked-MEPS from the M1 across 24 h time course**. MEP values at each timepoint are averaged. Error bars indicate SEM. Asterisks indicate significant differences.

With regard to the application of CBZ, there was a significant main effects of DRUG + tRNS condition [*F*_(1, 14)_ = 4.98; *p* = 0.007] and factor TIME [*F*_(10, 14)_ = 2.09; *p* = 0.03]. CBZ showed a tendency toward inhibiting MEPs between 5–60 and 120 min poststimulation (*p* < 0.05) (Figure 1). The effect LOR on MEPs was significant for the interaction of DRUG × TIME [*F*_(10, 14)_ = 1.85; *p* = 0.05] (Figure 4). The *post-hoc* test demonstrated significant differences at 0–20 (*p* < 0.01) and 60 min time points (*p* < 0.05) between the tRNS + PLC and tRNS + LOR conditions.

**Figure 2:**
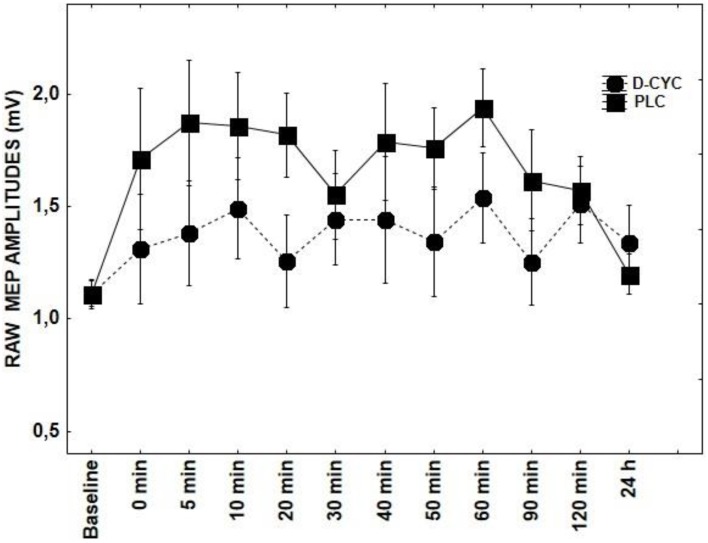
**Shows the effect of D-CYC on tRNS-induced aftereffects as measured by averaging TMS-evoked-MEPS from the M1 across 24 h time course**. MEP values at each timepoint are averaged. Error bars indicate SEM.

**Figure 3:**
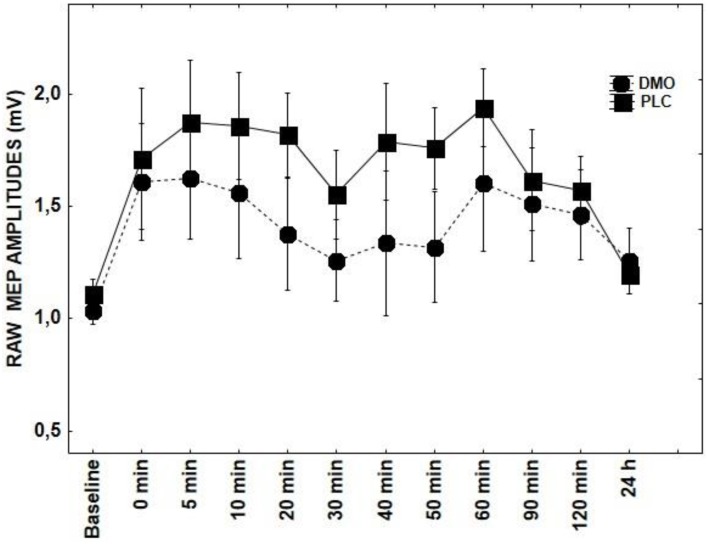
**Shows the effect of DMO on tRNS-induced aftereffects as measured by averaging TMS-evoked-MEPS from the M1 across 24 h time course**. MEP values at each timepoint are averaged. Error bars indicate SEM.

**Figure 4:**
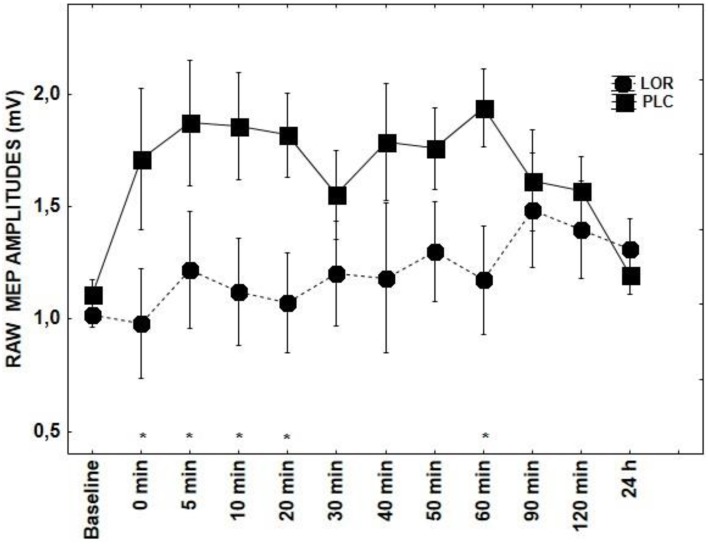
**Shows the effect of LOR on tRNS-induced aftereffects as measured by averaging TMS-evoked-MEPS from the M1 across 24 h time course**. MEP values at each timepoint are averaged. Error bars indicate SEM. Asterisks indicate significant differences.

**Figure 5:**
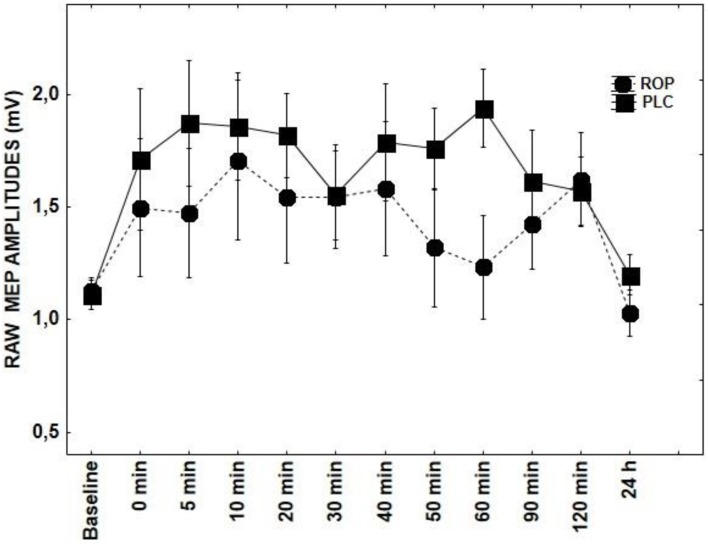
**Shows the effect of ROP on tRNS-induced aftereffects as measured by averaging TMS-evoked-MEPS from the M1 across 24 h time course**. MEP values at each timepoint are averaged. Error bars indicate SEM.

## Discussion

By using a combination of CNS active drugs in conjunction with single-pulse TMS we were able to partially profile the receptor mechanisms underlying tRNS-induced neuroplasticity. We tested the involvement of GABA_*A*_ (LOR) and D_2_/D_3_ receptors (ROP) in addition to NMDA receptor blockade or potentiation (DMO and D-CYC). Sodium channel conductivity was also investigated with the application of CBZ. Results indicate that tRNS induced plasticity is probably NMDA receptor independent, as administration of DMO showed no blocking effect on tRNS-induced excitability increases, although a light tendency toward inhibition was seen. D-CYC, a partial NMDA receptor agonist also showed no effect on tRNS aftereffects, in contrast to the expected prolongation of aftereffects (Nitsche et al., 2004a). The main result of this study is that CBZ, a voltage gated sodium channel blocker, significantly curtailed the excitability enhancing effect of tRNS, normally lasting up to 60 min. Furthermore, administration of LOR showed a trend toward reducing the increase in cortical excitability seen after application of tRNS, which was most marked at the 0–20 and 60 min post-stimulation.

Our data also suggests no role of dopaminergic modulation for tRNS, as ROP in a dosage of 2 mg did not have an effect on tRNS-induced aftereffects. In a comparable tDCS study ROP produced a biphasic response, where low (0.125 and 0.25 mg) and high (1.0 mg) dosages impaired both cathodal and anodal tDCS-induced neuroplasticity, compared with the placebo condition (Monte-Silva et al., 2009). Under medium dosage (0.5 mg), ROP did not influence the anodal tDCS-elicited aftereffects. In contrast, a prolonged inhibition was observed in the cathodal tDCS condition. ROP in a dosage of 2 mg was not tested in this study. In another study low dose pergolide didn't affect anodally-induced MEP increases either; only the duration of cathodal excitability diminutions was clearly prolonged (Nitsche et al., 2006). This result does not, however, exclude an influence of the dopaminergic system, since the interaction between either L-Dopa or dopamine agonists and transcranial stimulation is dose-dependent and complex (overview in Nitsche et al., 2010).

The mechanisms underlying the inductive excitatory aftereffects of tRNS are as yet unknown, and have been postulated to be modulated through the potentiation of voltage- gated sodium channels (Terney et al., 2008). The application of tRNS, a repetitive subthreshold electrical stimulation applied to the intact cortex, may alter the activation of voltage-gated sodium channels (Bromm, 1968). A continual influx and efflux of ions across the neuronal membrane has a high likelihood of being influenced by random AC current. Repeated depolarization may, for example, generate a cumulative cyclic response of sodium channels to continually repolarise and depolarise, and in this fashion may produce a heightened effect of the tRNS, resulting in the classical increases in cortical excitability observed in studies reporting tRNS-induced neuroplastic effects in the M1 (Terney et al., 2008; Chaieb et al., 2009). The temporal summation of these weak depolarising currents at the neuronal level may in turn, enhance the communication between neurons firing at the same rate and so may contribute to LTP-like changes, resulting in e.g., an enhancement in cognitive performance at the behavioral level (Fertonani et al., 2011; Cappelletti et al., 2013; Miniussi et al., 2013; Snowball et al., 2013). Similar responses to repetitive high frequency stimulation have also been observed in rodent electrophysiological preparations, where a weak depolarization of the neuronal membrane in cultured rat neurons could be observed after the application of extracellular stimulation (Schoen and Fromherz, 2008). The observation in this study that tRNS aftereffects were reduced by administration of CBZ, a sodium channel antagonist, suggests that tRNS-induced plasticity effects may be indeed sodium channel dependent. The “leaky” nature of sodium channels at subthreshold conductances, due to the ability of sodium channels to rapidly recover from inactivation during depolarization, means that a resurgent current upon repolarization is present in some neuronal populations (Raman and Bean, 1997; Grieco et al., 2005). This may be of importance since sodium channels are one of the most abundant voltage-gated ion channels present on the cell membrane (Yu and Catterall, 2003). The interaction between the membrane-modulating effects of electrical currents and CBZ has also been investigated using tDCS. CBZ administration resulted in the abolishment of anodal stimulation induced increase in cortical excitation but did not have any blocking effect upon cathodally induced excitability decreases (Liebetanz et al., 2002; Nitsche et al., 2003a).

In addition to the blocking effect of CBZ, LOR, a GABA_*A*_ receptor agonist, also influenced the amplitude of TMS-evoked-MEPs after tRNS. The reduction in MEP amplitude started immediately post-stimulation (0 min) to 20 min. This short effect may be attributed to the relatively small dosage of LOR administered to the participants. It may well be that at higher dosages (2 mg, instead of the 1 mg administered here), LOR may have a more pronounced effect of tRNS-induced aftereffects, although at much higher doses unwanted side effects are also exacerbated (Izaute and Bacon, 2005). We do not know yet to what extent tRNS is affecting or influencing cortical rhythmicity, which on a macroscopic level would be an amplification of its more direct effects on the potentiation of ion channels at the membrane. Even though the effect of LOR on tRNS-induced neuroplastic aftereffects was not significant, we were still able to see a tendency toward a reduction of tRNS aftereffects.

As a result of the medication, none of the subjects complained of dizziness, vertigo or fatigue. None of the experiments had to be terminated due to side effects. Therefore, the blinding of subjects or the person conducting the experiments was completely valid during these measurements.

One of the limitations of this work is the small number of subjects. The negative or small results might be related to the number of subjects (*n* = 8), although the sample size calculations based on previous results suggested that this number of subjects should be sufficient. Indeed, previous studies recruited even less subjects testing the effect of transcranial stimulation methods using the same or similar drugs (e.g., Huang et al., 2007). Increasing the sample size might result in more weighty changes (e.g., the main effect of TIME was significant by several drugs, nevertheless the DRUG × TIME interaction was not). Nevertheless, this study was designed as a pilot study providing first efficacy data. It should be noted that these results apply only to the dosages used, and to the parameters tested in this study. Further experiments should also control the level of attention that can be substantially altered by drugs (e.g., LOR) and might also affect tRNS-induced plasticity.

## Conclusion

Although most neuroplasticity induction mechanisms of tES methods are thought to be mediated by NMDA-receptor potentiation, this was not the case for our study (for a review on tES see Paulus, 2011). We observed a more pronounced effect of voltage-gated sodium channels on tRNS aftereffects. However, the neuroplasticity-inducing effects of oscillating currents may lie within the modulation of ion channels located on the neuronal membrane, but may also affect ongoing cortical rhythmicity as observed on the behavioral level (Fertonani et al., 2011). It may appear that even though tRNS is subject to membrane modulation effects, its potential role in interfering with ongoing cortical rhythmicity is not mutually exclusive.

### Conflict of interest statement

The authors declare that the research was conducted in the absence of any commercial or financial relationships that could be construed as a potential conflict of interest.
